# Surface morphology and optical properties of porphyrin/Au and Au/porphyrin/Au systems

**DOI:** 10.1186/1556-276X-8-547

**Published:** 2013-12-27

**Authors:** Yevgeniya Kalachyova, Oleksiy Lyutakov, Andrey Solovyev, Petr Slepička, Vaclav Švorčík

**Affiliations:** 1 Department of Solid State Engineering, Institute of Chemical Technology, Prague 166 28, Czech Republic; 2 Institute of Chemical Process Fundamentals of the AS CR, Prague 165 02, Czech Republic

**Keywords:** Nanostructures, Gold, Porphyrin, Luminescence, Enhancement, Surface morphology

## Abstract

Porphyrin/Au and Au/porphyrin/Au systems were prepared by vacuum evaporation and vacuum sputtering onto glass substrate. The surface morphology of as-prepared systems and those subjected to annealing at 160°C was studied by optical microscopy, atomic force microscopy, and scanning electron microscopy techniques. Absorption and luminescence spectra of as-prepared and annealed samples were measured. Annealing leads to disintegration of the initially continuous gold layer and formation of gold nanoclusters. An amplification of Soret band magnitude was observed on the Au/meso-tetraphenyl porphyrin (TPP) system in comparison with mere TPP. Additional enhancement of luminescence was observed after the sample annealing. In the case of sandwich Au/porphyrin/Au structure, suppression of one of the two porphyrins’ luminescence maxima and sufficient enhancement of the second one were observed.

## Background

Thin, discontinuous metal films with an island-like structure have attracted large scientific and practical interest due to their specific properties and multiple applications based on the surface plasmon resonance phenomenon. Surface arises from the interaction of light with free electrons at the dielectric/metal interface. The position and width of the plasmon resonance peak depend on the size and shape of the metal particles and their environment [1,2]. Surface plasmon resonance is used in various sciences and technology fields, e.g., as highly sensitive chemo- and biosensors [3]. Additionally, enhancement of the electromagnetic field at the metal/dielectric interface [4] is responsible for surface-related nonlinear optical phenomena [5] such as surface-enhanced Raman scattering (SERS), second harmonic generation [6], enhanced absorption [7], and surface fluorescence (SEF) [8].

SERS and SEF arise from the amplification of the response of an analyte molecule deposited near or on a roughened metal substrate. There are two theories as to the origin of the surface-enhanced phenomena. According to the first one, the enhancement is mainly due to the amplified electromagnetic field at the metal surface [9-11]. The second one ascribes the enhancement to chemical enhancement, where metal/molecule charge transfer complexes are formed and enrich resonance with the excitation laser [12].

Flat metallic films generally have very small effects on the SEF or SERS phenomena. However, by increasing the surface roughness, the cross sections of organic molecules deposited on the gold surface can be dramatically enhanced [13]. The linear and nonlinear optical properties of molecules deposited onto metallic films are affected by film surface roughness [14]. The largest enhancement was observed on molecules adsorbed on roughened surfaces comprising nanosized objects.

This work focuses on the study of luminescence activity of porphyrin deposited on nanostructured gold films. The origin of these phenomena is largely due to an enhanced electromagnetic (EM) field at the metal substrate surface due to photon-plasmon conversion [15-17].

## Experimental

### Materials

Meso-tetraphenyl porphyrin (TPP) of 99.7% grade was purchased from Frontier Scientific (Logan, UT, USA), and 99.99% pure gold target was supplied by Goodfellow Ltd. (Cambridge, UK). No additional purification of these materials was performed.

### Sample preparation

**Figure 1:**
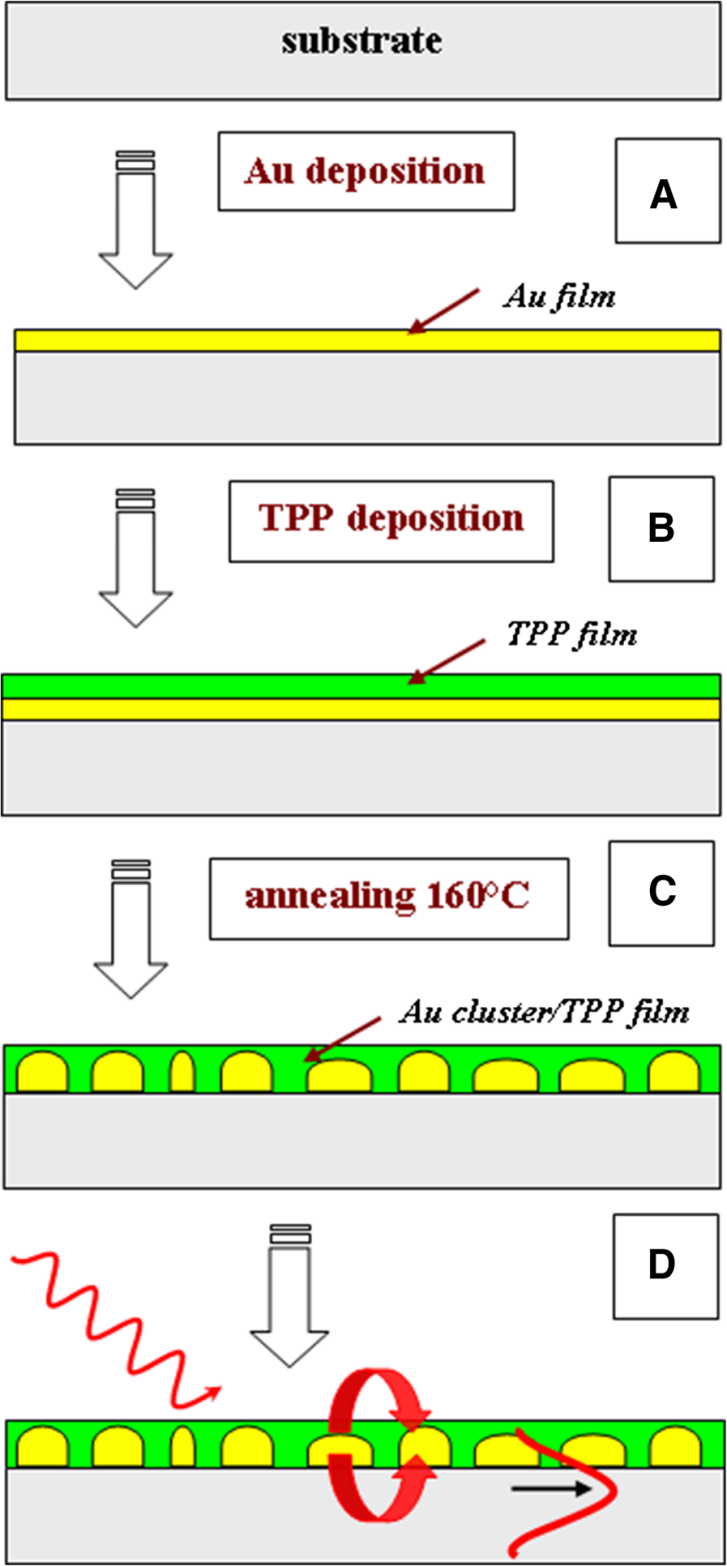
**Schematics of gold clustering and luminescence enhancement. (A)** Gold sputtering, **(B)** porphyrin evaporation, **(C)** temperature annealing and gold clustering, and (D) excitation of plasmon resonance with luminescence enhancement.

Multifilms of porphyrin and gold have been prepared on a glass substrate. The gold layers were sputtered on a microscopic glass (Glassbel Ltd., Prague, Czech Republic). The sputtering was accomplished on a Balzers SCD 050 device (Micronova, Espoo, Finland) under the following deposition conditions: DC Ar plasma, gas purity 99.995%, discharge power 7.5 W, sputtering time 25 s. Under these experimental conditions, a homogeneous distribution of gold over the glass surface was achieved [18]. Porphyrin layers were deposited by vacuum evaporation technique under 10^-6^-Torr pressure with 10-nm min^-1^ deposition rate. Post-deposition annealing of the Au-covered glass was carried out in air at 160°C for 24 h using a thermostat Binder oven. The heating rate was 5°C min^-1^, and the annealed samples were left to cool in air to room temperature. The method of the sample preparation is illustrated in Figure 1.

### Diagnostic techniques

Optical and confocal images of the samples’ surface were taken using the optical microscope Lext OLS 3100 (Olympus Corporation, Shinjuku, Tokyo, Japan). The surface morphology and roughness of the samples were examined by atomic force microscopy (AFM) on a Digital Instruments CP II Veeco device (Plainview, NY, USA), working in tapping mode with RTESPA-CP probes. The thickness of the prepared structures was measured by AFM-scratch method [19].

UV/Vis spectra were measured using UV/Vis Spectrometer Lambda 25 (PerkinElmer, Waltham, MA, USA). Photoluminescence spectra (excitation wavelength 440 nm) were obtained using the fluorescent spectrophotometer SPECTRA star Omega (BMG LABTECH GmbH, Ortenberg, Germany).

Sample cuts for scanning electron microscope (SEM) imaging were prepared by focused ion beam (FIB) method on an adapted SEM (FIB-SEM, LYRA3 GMU, Tescan, Czech Republic). The FIB cuts were made with a Ga ion beam, and the SEM images were taken under the angle of 54.8°. The influence of the angle on the images was automatically corrected by the SEM software. Polishing procedure was applied to clean and flatten the investigated surfaces.

## Results

### Structure of Au/TPP

The luminescence enhancement of porphyrin deposited onto the nanostructured gold surface was studied. Gold as a substrate and porphyrin as a probe molecule were chosen for the following reasons. Porphyrin is an organic dye with a larger extinction coefficient and highly efficient luminescence [11,20], and gold is the commonly used substrate for SERS applications. Gold nanostructures show unique properties due to localized surface plasmon oscillation in the Vis-NIR region [21]. The effect of the surface plasmon oscillation of gold nanoparticles on excitation of porphyrin molecules bound at the gold surface is quite interesting [22,23].

**Figure 2:**
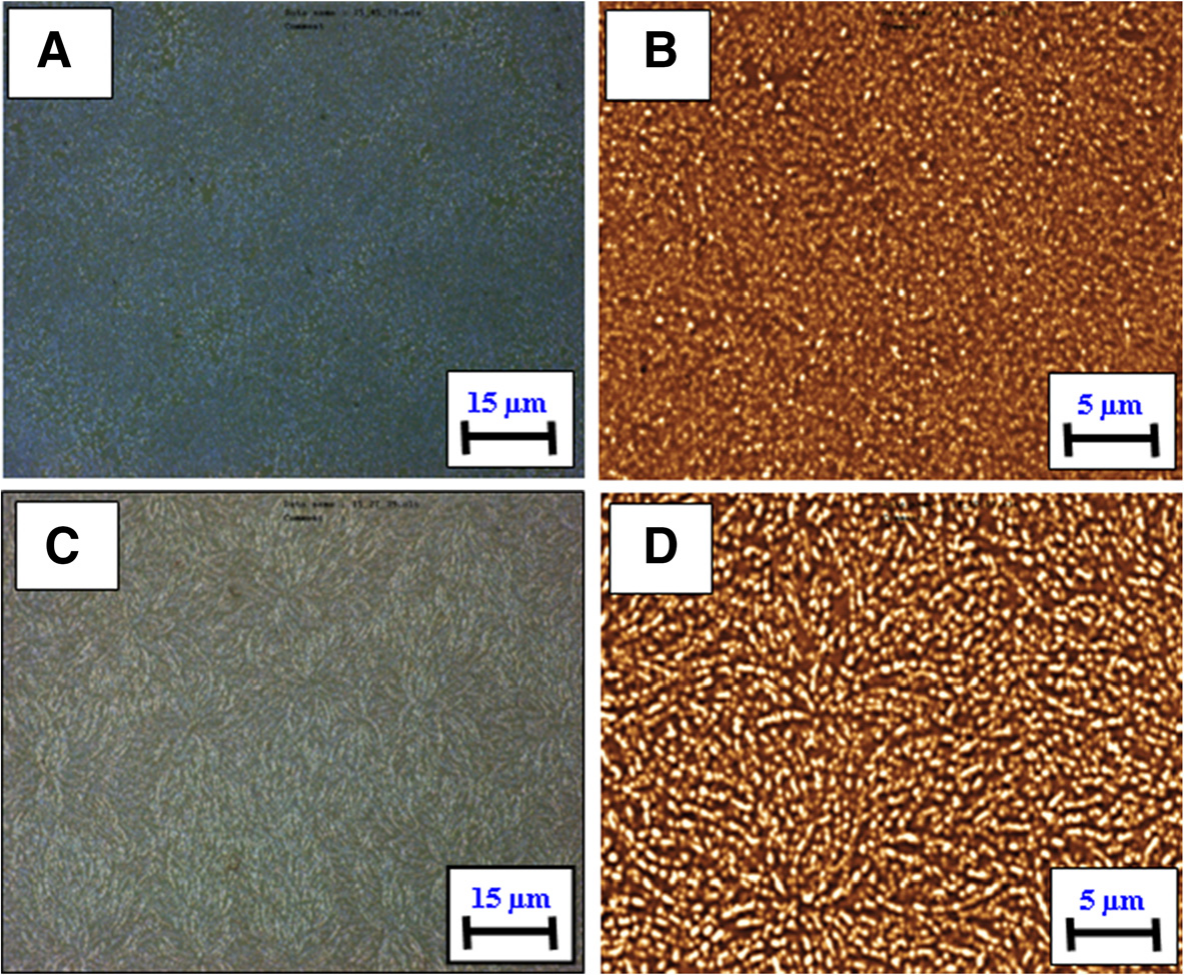
**Optical and confocal images of Au/TPP films deposited on glass.** Before **(A, B)** and after annealing at 160°C for 24 h **(C, D)**.

The gold layer (25 nm thick) was deposited on glass by vacuum sputtering, and then the porphyrin layer (50 nm thick) was evaporated onto the gold film. The samples were annealed at 160°C to initiate gold clustering and to produce a nanostructured Au/TPP system. Changes in the surface morphology were analyzed by optical microscopy, confocal microscopy, and AFM. Optical and confocal images of the Au/TPP film taken before annealing are shown in Figure 2A,C and those taken after annealing in Figure 2B,D. Significant changes of the surface morphology after annealing are evident. The sample surface becomes rougher and an island-like structure arises. Initially, flat gold layers disintegrate into a system of randomly distributed gold clusters with various sizes and shapes. Such behavior of thin gold films under annealing is well known and was repeatedly described [24,25]. In our case, the created gold clusters represent a random ensemble of gold nanoparticles with characteristic surface plasmon resonance and related absorption band.

**Figure 3:**
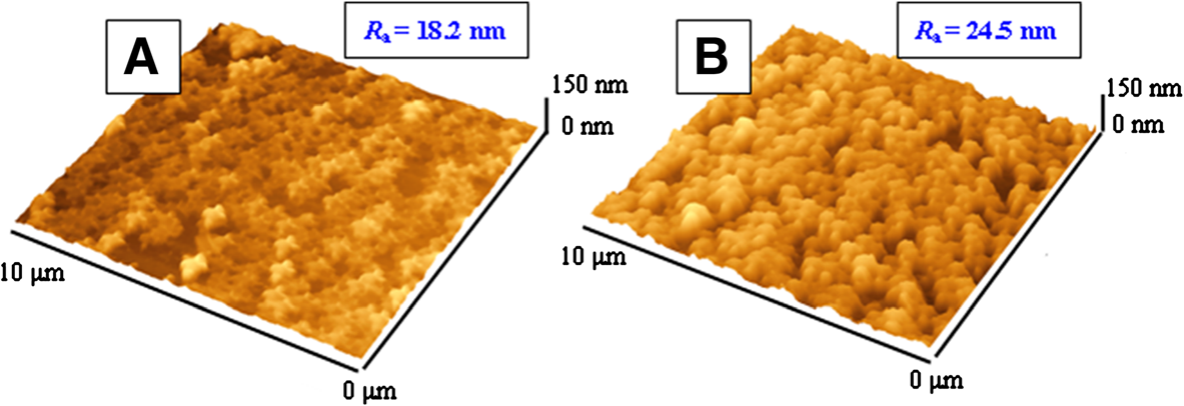
AFM of Au/TPP films deposited on glass before (A) and after annealing at 160°C for 24 h (B).

Additional information on surface morphology was obtained using the AFM technique. Typical surface morphologies of Au/TPP films observed before and after annealing are shown in Figure 3 together with the measured surface roughness *R*_a_. After annealing, rather homogenously distributed bulges appear on the sample surface which may represent crystal aggregates of porphyrin molecules formed onto the gold surface during porphyrin evaporation (see Figure 3B). For better characterization of the surface morphology, a quantitative analysis of AFM scans was performed. The cluster size and distribution were determined using SPM Lab Analysis software and approximated by Gaussian distribution. Results are given in Table 1.

**Table 1:** Results of surface analysis from AFM measurements (Gaussian approximation) of pristine and annealed Au/TPP and Au/TPP/Au structures

**Sample**	**Cluster**	**Maximum peak**	**Half-width of maximum**
Pristine Au/TPP	Height (nm)	61.0	21.0
	Perimeter (μm)	4.0	1.2
Annealed Au/TPP	Height (nm)	51.0	31.0
	Perimeter (μm)	5.4	2.1
Pristine Au/TPP/Au	Height (nm)	15.1	7.5
	Perimeter (μm)	2.7	0.4
Annealed Au/TPP/Au	Height (nm)	27.2	14.3
	Perimeter (μm)	3.2	0.9

**Figure 4:**
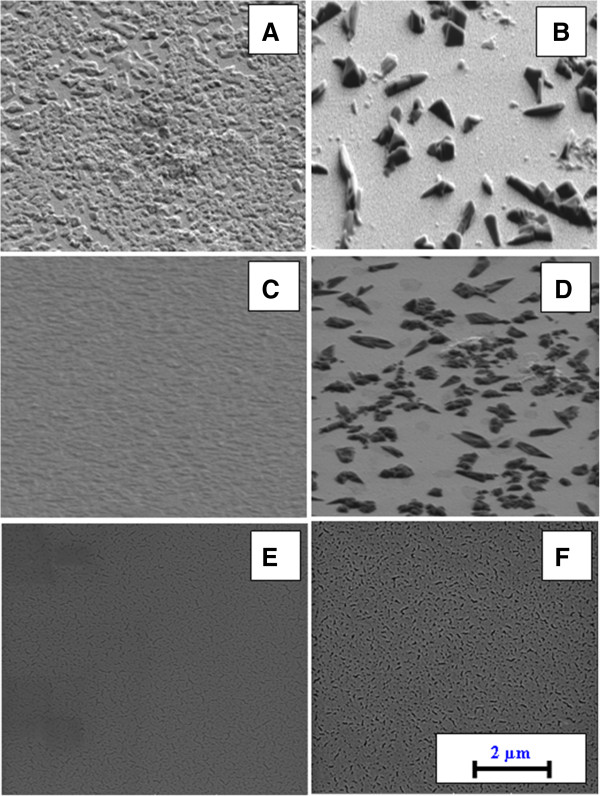
**SEM images of structures before and after annealing at 160°C for 24 h.** Au/TPP/glass **(A, B)**, Au/TPP/Au/glass **(C, D)**, and Au/glass **(E, F)**.

This surface evolution is initiated by the tendency of the thin gold film to form randomly distributed island-like structures under annealing. In this case, surface morphologies of annealed pure Au [24] and Au/TPP films are quite similar. Annealing at a given temperature obviously results in phase transition of gold films and disintegration of initially flat films into a system of randomly ordered clusters [26]. There are several mechanisms concerning gold film clustering and reported in the literature. As one example, capillary instabilities in thin, continuous films can be responsible for gold agglomeration [27]. In [28], Au clustering was attributed to gold island diffusion on the glass surface. The activation energy and diffusion coefficient for island mobility were found to be of the same order of magnitude as those for single-atom surface diffusion. A more plausible and intuitive explanation consists in the reduction of the surface energy of the system of ‘small’ gold clusters by their agglomeration [29]. However, in general, the exact mechanism of gold disintegration is not clear. Results of AFM studies were verified by SEM. Figure 4 shows SEM images of the surface of Au/TPP films before and after annealing. Changes of surface morphology during thermal treatment are evident from Figure 4A,B. Additionally, pure Au films before and after annealing are also shown (Figure 4E,F).

**Figure 5:**
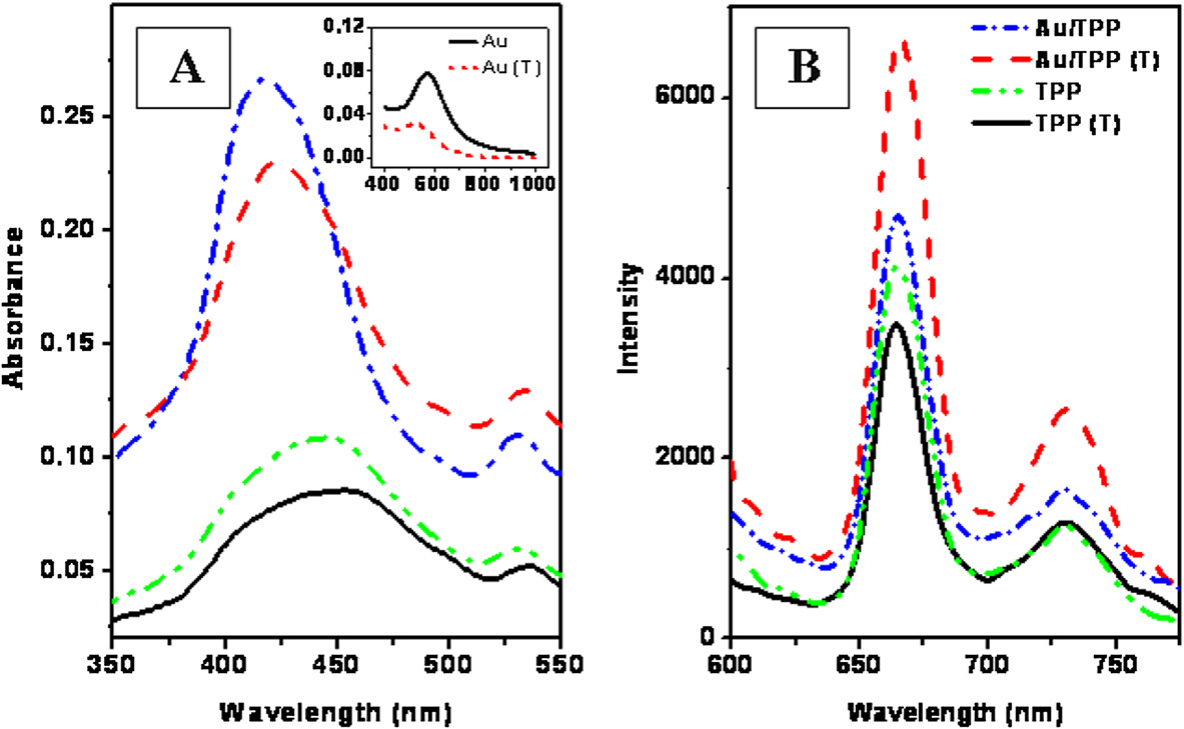
Absorption (A) and luminescence (B) spectra of Au/TPP films on glass before and after annealing (T).

The absorption and luminescence spectra of Au/TPP films before and after annealing are shown in Figure 5 and compared with the absorption and luminescence spectra of mere TPP layer deposited onto glass substrate. The absorption spectra of Au before and after annealing are shown in Figure 5A inset. In the absorption spectra of TPP and Au/TPP structures, the so-called Soret band is clearly visible. This absorption band achieves its maximum at 440 nm. In both cases, TPP and Au/TPP, the Soret band becomes slightly less intense after annealing. Probably, TPP molecules tend to aggregate during annealing and the aggregation reduces porphyrin-light interaction. More pronounced differences were observed between TPP and Au/TPP absorption spectra. An apparent amplification of Soret band magnitude was observed on the Au/TPP structure in comparison with mere TPP layer. This phenomenon cannot be explained by only addition of Au and TPP layer absorption.

Because the maximum of absorption peak lies at 440 nm, this wavelength was chosen for luminescence excitation. Figure 5B shows the porphyrin luminescence spectra of TPP and Au/TPP before and after annealing. Two luminescence maxima are seen at 660 and 730 nm. These maxima arise from singlet-singlet electron radiative transition and correspond to TPP’s two vibration states. After annealing, the luminescence of the TPP layer decreases slightly. The luminescence intensity of Au/TPP is higher than that of mere TPP layer. After annealing, the difference between TPP and Au/TPP luminescence spectra becomes more pronounced (the intensity increases twice).

### Sandwich film

**Figure 6:**
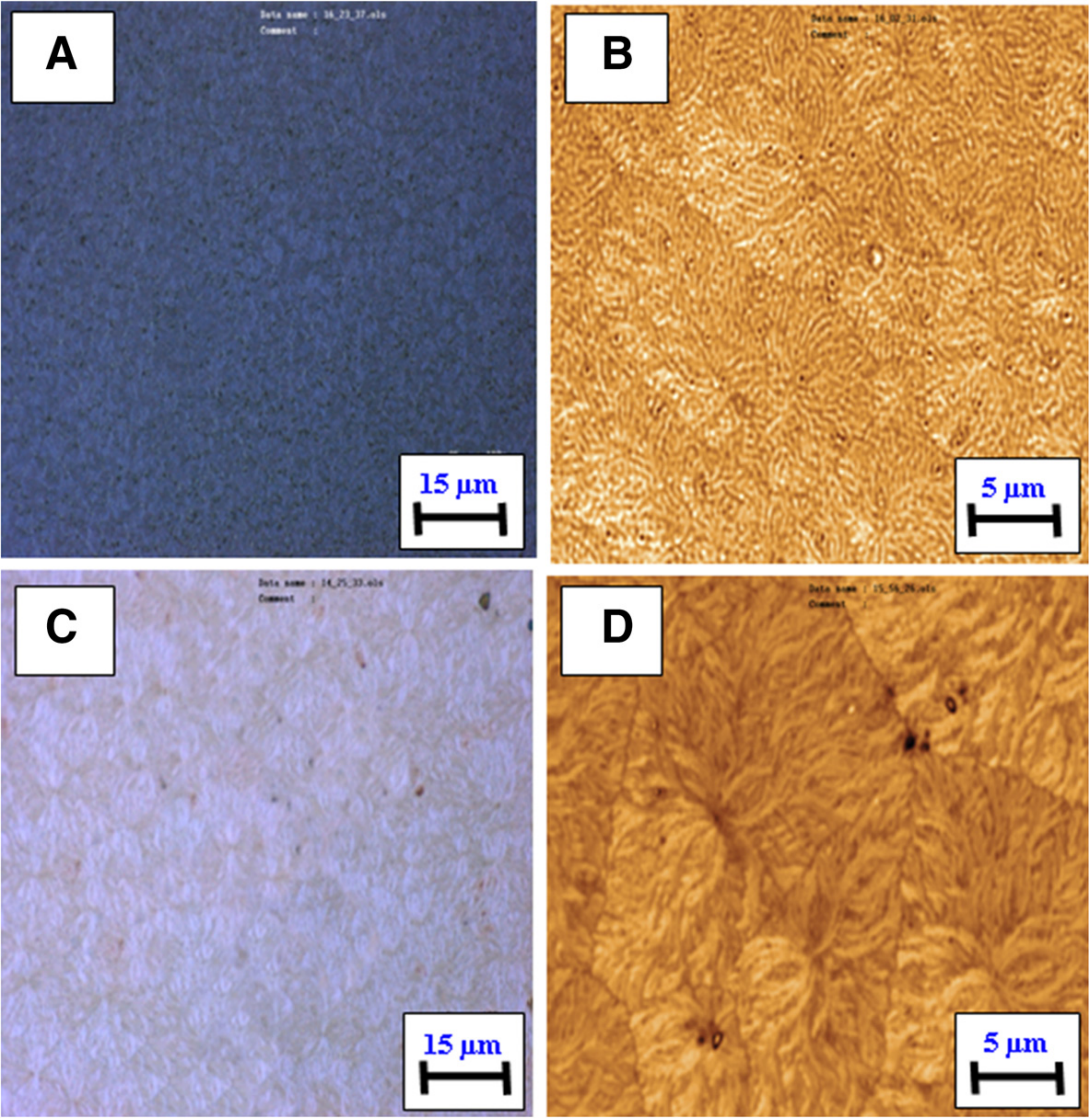
Optical and confocal images of Au/TPP/Au films deposited on glass before (A, B) and after annealing for 24 h (C, D).

Sandwich structures were prepared by gradual deposition of Au, TPP, and Au. After preparation, these structures were also annealed to achieve Au clustering. The surface morphology of these structures before and after annealing was determined by optical microscopy and AFM, and the typical images are shown in Figure 6. One can see that annealing leads to sufficient changes in the surface morphology. The supposed diffusion of gold atoms leads to disintegration of the initial multilayer structure.

**Figure 7:**
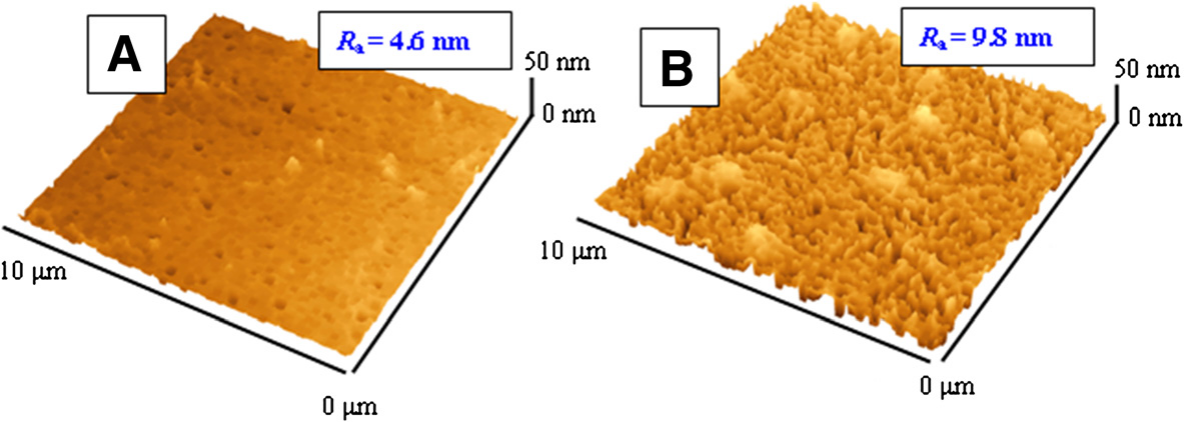
**AFM of Au/TPP/Au and TPP films deposited on glass.** Before **(A)** and after annealing (T) at 160°C for 24 h **(B)**.

**Figure 8:**
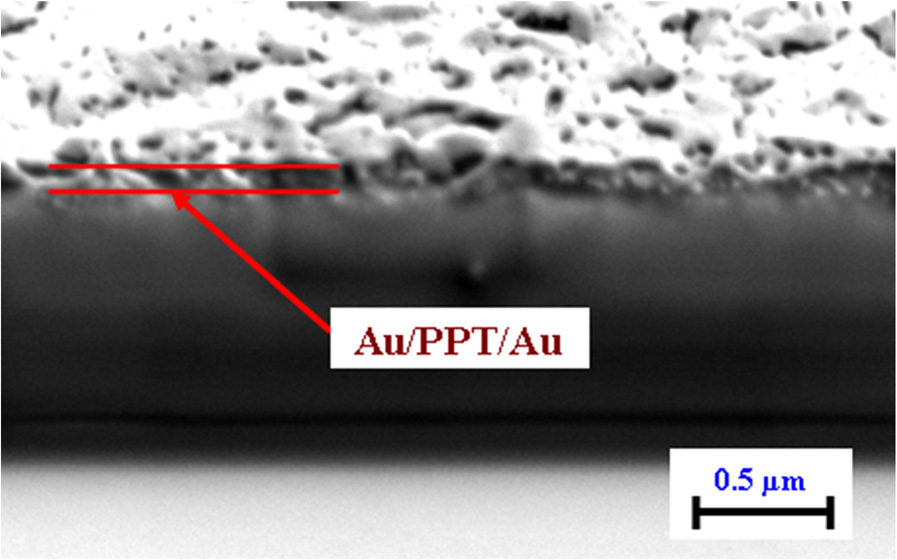
FIB-SEM image of the cross section of the Au/TPP/Au/glass structure taken under an angle of 54.8°.

The typical AFM images of Au/TPP/Au multifilms taken before and after annealing are shown in Figure 7. A nanostructured, random-ordered surface is well visible in Figure 7B. So, AFM measurement confirms changes in the surface morphology which are also seen from an increase of the surface roughness *R*_a_ from 4.6 to 9.8 nm. For better characterization of surface morphology, a quantitative analysis of AFM scans was also performed. Results are given in Table 1. Additional analyses of Au/TPP/Au structures by the SEM technique were also performed before and after annealing (Figure 4C,D). SEM images confirm AFM results, namely the increase of film roughness after annealing and the smoother surface of the Au/TPP/Au structure in comparison with the Au/TPP one. Additionally, the cross section of sandwich films was measured by the FIB-SEM technique (Figure 8). In this case, however, it is slightly difficult to identify the sandwich structure of the sample unambiguously.

**Figure 9:**
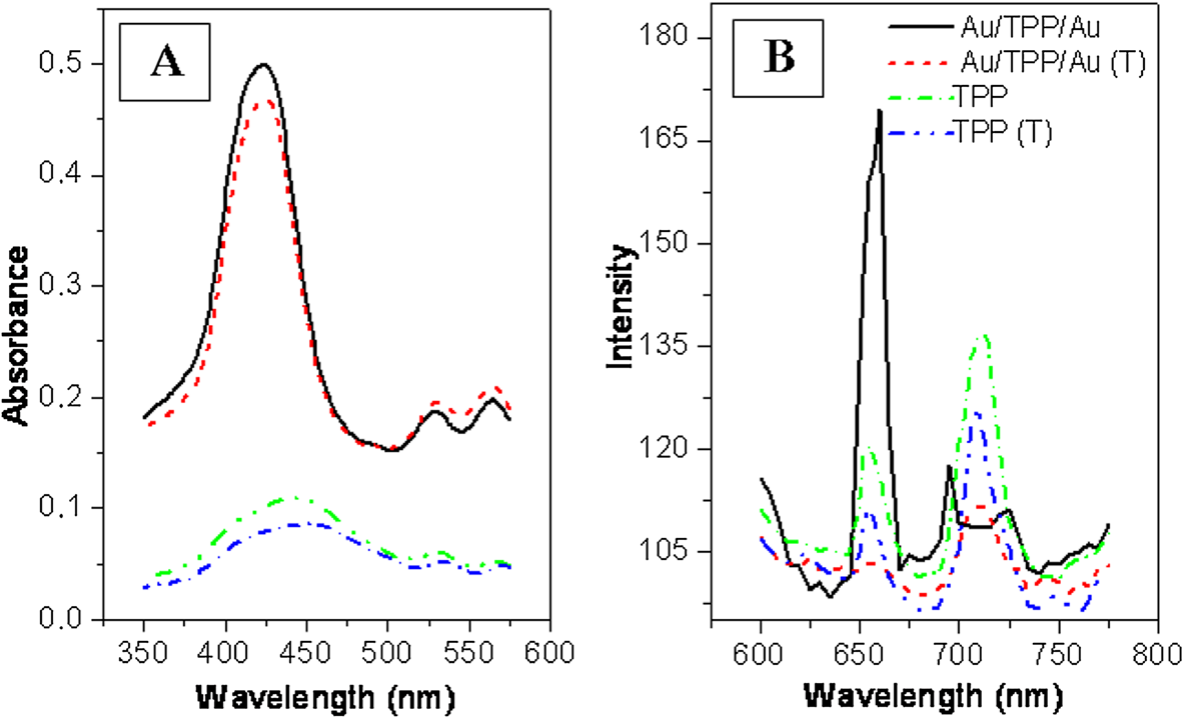
Absorption (A) and luminescence (B) spectra of Au/TPP/Au and TPP films annealed (T) at 160°C for 24 h.

The absorption and luminescence spectra of Au/TPP/Au and TPP films taken before and after annealing are shown in Figure 9. From Figure 9, it is evident that the annealing of TPP leads to an absorption peak reduction. As in the previous case, the combination of TPP with Au results in the appearance of the Soret band. Figure 9B shows the luminescence spectra excited at 440 nm. A principally different result was obtained in the case of the sandwich Au/TPP/Au structure in comparison with Au/TPP. In the former case, the luminescence peak at 720 nm is almost completely suppressed but another peak at 660 nm increased significantly. After annealing, a luminescence quenching was observed.

## Discussion

### Au/TPP structure

The Soret band increases several times after TPP deposition onto the gold surface. The phenomenon cannot be explained by only the presence of Au and TPP components. Similar phenomena, i.e., a luminescence increase, were reported earlier for a mixture of dyes with colloid metal nanoparticles [30]. In this case, the luminescence intensity increased twice. The absorption and luminescence increase can be explained in terms of photon-plasmon conversion. Excitation of plasmons leads to a sufficient light energy concentration near the gold surface, where TPP molecules are located. As a result, more energy is absorbed and re-emitted. On the other hand, absorption increases several times, but luminescence is only doubled. The missing part of the absorbed energy is probably expended through nonradiative relaxation of the excited state. This luminescence quenching becomes notable due to the proximity of the Au surface. The quenching is a result of a very strong nonradiative energy transfer from chromophores to the metal substrates. This effect is typical for a dye deposited primarily onto a metal surface and can be overcome by addition of a thick intermediate layer [31].

Assembled molecular layers of porphyrin derivatives are often created by the Langmuir-Blodgett (LB) method [32]. Another method consists in covalently binding of porphyrins to a gold surface through Au-S interactions [33,34]. Highly ordered adlayers of porphyrin molecules were found to form on a sulfur-modified Au (111) surface in [35]. Different orientations were achieved depending on the number of thiol groups per porphyrin molecule: porphyrin molecules having a single chain are somewhat tilted against surface normal, and porphyrins with four chains are oriented coplanar. Spacer length also affects the orientation of porphyrins onto the gold surface - as the length of spacers increases, porphyrin molecules tend to form highly ordered structures on the gold surface [36]. The obtained results indicate the dependence of porphyrin orientation and degree of gold surface covering on the crystal orientation of gold, quality of gold surface, and type of porphyrin used. Several porphyrins were also deposited from the vapor phase onto a gold surface. In the case of TPP, the molecules are preferentially oriented with the porphyrin ring parallel to the gold surface [37].

### Sandwich film

Comparison of the surfaces of Au/TPP and Au/TPP/Au before annealing indicates that the surface of Au/TPP/Au is more flat than that of Au/TPP. A possible explanation consists in the flattening of roughening of the Au/TPP surface during deposition of additional layer of Au. Probably, Au atoms migrate on the surface after contact with the substrate and tend to stand in the region of ‘valley’, which leads to surface smoothening.

Enhancement of the Soret band occurs in the case of the sandwich Au/TPP/Au system. This phenomenon is of similar nature to the case of Au/TPP films, and it is related to photon-plasmon conversion. However, in this case, a suppression of one of the two luminescence maxima in luminescence spectra is evident. According to the semi-classical Franck-Condon principle, two luminescence peaks appear due to transition of excited energy from the TPP’s lowest vibration excited state to two vibration states of TPP in the ground state. When TPP is sandwiched between Au layers, one of these radiative transitions is suppressed and the second luminescence peak increases approximately twice. It indicates that the excited TPP molecule can return to only one vibration ground state. We propose that one of the TPP’s vibration states is partially forbidden due to space confinement of the TPP layer by Au layers.

Comparison of the luminescence spectra of Au/TPP and Au/TPP/Au indicates weaker luminescence in the case of Au/TPP/Au. A possible explanation consists in particular screening of active porphyrin layer by additional gold layer. The screening can affect both the intensity of incident beam from the light source and the intensity of luminescence light passing the detector.

As to luminescence quenching occurring after annealing, we propose elimination of porphyrin from Au structures during annealing. In this case, the top and bottom Au layers coalesce each other and exclude porphyrin molecules. As a result, nonradiative relaxation of the porphyrin excited state becomes dominant, due to mutual aggregation of porphyrin molecules and their interaction with gold clusters.

Optical properties of porphyrins depend strongly on the deposited molecule’s orientation relative to the substrate. Photophysical properties of deposited porphyrins depend on surface plasmon resonance occurring in gold structures [38]. In the case of covalently bound porphyrins, luminescence quenching generally occurs and depends on the spacer between porphyrin and gold [39]. Additionally, quenching of luminescence depends on the particle size and shape in the case of porphyrin attachment to gold nanoparticles [40]. The position of the porphyrin fluorescence peak can be affected by combination with noble metals [41,42]. In [43], an attachment of porphyrin to gold clusters through a molecular spacer was reported resulting in suppression of the quenching of the porphyrin excited singlet, as compared to the quenching of self-assembled porphyrins on a two-dimensional flat gold surface. In most reported works, the case of a ‘monomolecular’ adlayer of porphyrin was considered. According to our previously reported results, as-deposited gold films have a semi-crystallic nature, with several detectable crystallographic orientations. During annealing, due to a phase transition followed by atom rearrangements, the crystallographic orientation Au (111) becomes preferable [44]. On the other hand, we deal with porphyrin layers that are sufficiently thicker than monomolecular film. So in our case, a dependence of the optical properties on mutual crystallographic orientation (coplanar or perpendicular orientation of the porphyrin), on the distance between the porphyrin and gold substrate, and/or on the shape of the gold nanoparticles is not assumed.

The prepared nanostructures exhibit interesting optical properties and have a promising potential for different applications in photonics, energy conversion, and analytical methods [45,46]. Combination of gold islands arises, whose sizes and optical properties can be controlled by subsequent annealing [47]. The gold with the deposited layer of porphyrin was used to enhance the resolution of optical spectroscopy. Gold-porphyrin films will found their application in light-harvesting systems for photocurrent generation [48]. These structures will also be useful in the reduction of molecular oxygen [33,49]. Another attractive application of gold-porphyrin nanosystems lies in the preparation of multibit information storage devices [50]. Additionally, gold electrodes modified by porphyrin or porphyrin-fullerene systems will be used for artificial photosynthesis [51,52]. Moreover, self-assembled porphyrins on Au surface can serve as enantioselective sensors or biosensors [53,54].

## Conclusions

The preparation of two different porphyrin/gold and gold/porphyrin/gold systems is described. A slight enhancement of the luminescence intensity was found in the case of the porphyrin/Au structure. Additional luminescence enhancement was observed after sample annealing. The enhancement is related to disintegration of the initially continuous gold film into an island-like structure and to excitation of surface plasmons. A sandwich gold/porphyrin/gold system with porphyrin intermediate layer was also studied. In this case, suppression of one of the two luminescence maxima and sufficient enhancement of the second one were observed.

## Competing interests

The authors declare that they have no competing interests.

## Authors’ contributions

YK carried out the sample preparation and modification. OL performed the interpretation of obtained results and coordination of the work AS participated in the optical measurements. PS carried out samples surface characterization. VŠ participated in the sample design and coordination. All authors read and approved the final manuscript.
